# Potential risks associated with the use of ionizing radiation for imaging and treatment of colorectal cancer in Lynch syndrome patients

**DOI:** 10.1007/s10689-022-00299-9

**Published:** 2022-06-20

**Authors:** Mingzhu Sun, Jayne Moquet, Michele Ellender, Simon Bouffler, Christophe Badie, Rachel Baldwin-Cleland, Kevin Monahan, Andrew Latchford, David Lloyd, Susan Clark, Nicola A. Anyamene, Elizabeth Ainsbury, David Burling

**Affiliations:** 1 UK Health Security Agency, Department of Radiation Effects, RCEHD, Chilton, Didcot, OX11 0RQ UK; 2 Intestinal Imaging Centre, St Mark’s Hospital, London North West University Healthcare NHS Trust, Watford Road, Harrow, HA1 3UJ UK; 3 Lynch Syndrome Clinic, Centre for Familial Intestinal Cancer, St Mark’s Hospital, London North West University Healthcare NHS Trust, Watford Road, Harrow, HA1 3UJ UK; 4 East and North Hertfordshire NHS Trust, Mount Vernon Cancer Centre, Rickmansworth Road, Northwood, HA6 2RN Middlesex UK; 5 Environmental Research Group Within the School of Public Health, Faculty of Medicine at Imperial College of Science, Technology and Medicine, London, W12 0BZ UK

**Keywords:** Lynch syndrome, DNA mismatch repair deficiency, Familial colorectal cancer, Ionizing radiation, Radio-sensitivity

## Abstract

The aim of this review is to investigate the literature pertaining to the potential risks of low-dose ionizing radiation to Lynch syndrome patients by use of computed tomography (CT), either diagnostic CT colonography (CTC), standard staging CT or CT surveillance. Furthermore, this review explores the potential risks of using radiotherapy for treatment of rectal cancer in these patients. No data or longitudinal observational studies of the impact of radiation exposure on humans with Lynch syndrome were identified. Limited experimental studies utilizing cell lines and primary cells exposed to both low and high radiation doses have been carried out to help determine radio-sensitivity associated with DNA mismatch repair gene deficiency, the defining feature of Lynch syndrome. On balance, these studies suggest that mismatch repair deficient cells may be relatively radio-resistant (particularly for low dose rate exposures) with higher mutation rates, albeit no firm conclusions can be drawn. Mouse model studies, though, showed an increased risk of developing colorectal tumors in mismatch repair deficient mice exposed to radiation doses around 2 Gy. With appropriate ethical approval, further studies investigating radiation risks associated with CT imaging and radiotherapy relevant doses using cells/tissues derived from confirmed Lynch patients or genetically modified animal models are urgently required for future clinical guidance.

## Introduction to Lynch syndrome

### Lynch syndrome and its genetic background

Lynch syndrome (LS), previously referred to as hereditary non-polyposis colorectal cancer (HNPCC), is the most common cause of hereditary colorectal cancer (CRC, approximately 2–5%) [1]. LS patients have an increased risk of CRC, adenomatous polyps and other extra-colonic malignancies [2]. The average age for LS patients developing CRC is 45 years; and in comparison it is 63 years for sporadic CRC in the average risk population [1]. Individuals with LS have a high cumulative lifetime risk of developing CRC (15%-70% at age 70) [3], which leads to a need for LS patients to be part of an effective surveillance program.

Genetically, LS is an inherited disorder characterized by constitutional pathogenic variants in the coding sequence or regulatory domains of the DNA mismatch repair (MMR) genes, most commonly *MLH1*, *MSH2*, *MSH6* and *PMS2* [4]. Over 450 germline alterations have been described for the MMR genes (www.insight-group.org). In Western Europe, approximately one million individuals have been estimated to be carriers of an MMR defect [5]. The general population prevalence in the United States (US), Canada, and Australia is estimated in 2017 to be 1 in 279 [6].

The majority of LS cases (approximately 70–85%) are caused by *MLH1* or *MSH2* mutations, whereas mutations in *MSH6* and *PMS2* each account for 10–20% of cases. Additionally, abnormality in an upstream non-MMR gene, *EPCAM*, may cause the repression of *MSH2* [2]. LS can also develop when there is a rare germline epigenetic modification of the *MLH1* promoter resulting in gene silencing [7]. Tumors demonstrating absent immunohistochemical (IHC) staining for any of the four MMR proteins are considered to have underlying dysfunction in the DNA MMR system as a result of either epigenetic, somatic, and/or germline MMR gene inactivation [8]. An MMR gene defect occurs through loss of corresponding normal alleles in the tumors of carriers resulting in loss of MMR function and subsequent accumulation of somatic mutations, detectable as microsatellite instability (MSI) in repetitive DNA segments called microsatellites [9].

### Diagnosis of LS

Features suggestive of pathogenic variants in the MMR genes may be identified by assessing the molecular phenotype of tumors relating to LS, such as MMR protein IHC or PCR-based MSI analysis. Universal screening of all colorectal and endometrial tumors with these analyses was recommended in 2018. Rapid and scalable somatic and germline sequencing using advanced next-generation sequencing technology now allow the identification of LS in individuals showing no classic phenotypes [8].

### Current surveillance of LS patients

For early detection and prevention of CRC, the European Society of Gastrointestinal Endoscopy recommends colonoscopy surveillance every two years. For *MLH1* and *MSH2* mutation carriers, the starting age for surveillance is 25 years, whereas for those carrying *MSH6* and *PMS2* variants, it’s 35 years. Currently, surveillance of other organs is not routinely offered as there are no data to support the benefit of this, and CT colonography is not recommended for large bowel surveillance even though it has several advantages over colonoscopy [3]. It is also worth noting that the true prevalence of MMR deficient (d-MMR) rectal cancer is not well established, although it is likely to be less common than for colon cancer, for either MMR status may influence oncological decision making (www.nice.org.uk/guidance/ta709).

### Treatment

For localized CRC, surgical resection is the primary treatment. Neoadjuvant radiotherapy is used routinely for patients with advanced rectal cancer, including those with lympho-vascular involvement and those whose tumors extend beyond standard surgical anatomical planes [10]. Currently, MMR status is not routinely considered prior to use of radiotherapy in this pre-operative setting, and yet vital information about the radio-sensitivity of both the tumor and its surrounding tissues is required to support such decision making.

In a recent review, it was stated that advanced/metastatic Lynch and non-Lynch cancers with MSI can be treated with anti-PD-1 (anti-programmed cell death protein 1) monoclonal antibodies (pembrolizumab or nivolumab). 70% or greater disease control rates have been achieved, many with long lasting effects [8]. Some LS patients with an MSI-High metastatic tumor now have long-term and even complete clinical responses to immunotherapy [11].

### Literature review

This review considers the literature associated with the potential radiation risks for LS patients. A comprehensive search of peer reviewed journals was carried out by a librarian in the following databases: Medline, PubMed, EMBASE and Google Scholar. A wide range and combinations of key terms were used including Lynch syndrome, HNPCC, mismatch repair, microsatellite instability, CT colonography, radiosensitivity, colorectal cancer, radiation, MLH1, MSH2, MSH6, and PMS2. Initially, over 300 papers were identified. The abstracts were then filtered to include only those on LS or MMR deficiency and their association with CT scan, radiation or radiotherapy. Further filtering was conducted by full-text reading for relevance to the topic. A total of 71 articles were referenced in this review.

## Radiation risk for CT and radiotherapy

Currently, there is a lack of evidence on the harm caused by radiation to LS patients, and no published guidance on the medical use of radiological imaging and radiotherapy for these patients.

### Low-dose radiation risk

Ionizing radiation (IR), such as X-rays used in diagnostic imaging and radiotherapy, can cause a wide range of direct and indirect DNA damage [12, 13]. The international unit of measure for absorbed radiation dose is the gray (Gy), defined as the absorption of one joule of radiation energy per kilogram of matter. The unit of sievert (Sv) is used to express the equivalent dose depending on source and properties of the biological target [14]. For low linear energy transfer electromagnetic radiations such as X-rays and γ-rays, the gray and sievert can be considered equivalent [13]. The average effective dose from CTC is approximately 8–10 mSv [15], and on average individuals receive 2.4 mSv of IR annually from natural sources [16]. Standard staging and surveillance body CT use a similar IR dose to CTC, whereas radiotherapy utilizes exponentially higher doses of radiation in the order of 25 Gy (short course over 5 days) or 45–50 Gy (long course over 5–6 weeks) [17].

There has been much debate about the risk of low-dose IR to the human population even though the linear no-threshold model, the current established method to estimate carcinogenic risk from radiation [18], suggests no threshold dose for radiation-induced malignancy based on the stochastic nature of radiation carcinogenesis [15]. Until this debate is ever resolved, the precautionary principle of linearity of dose response, with no safe low dose, is recommended and adopted by international consent [13]. Evidence for the association of low-dose exposure with increased death rate can be found in some long term studies involving large cohorts of workers [19, 20]. In 2016, the International Nuclear Workers Study reported the health effects of protracted low-dose exposure in nuclear industry workers (308,297) in France, the United Kingdom and the US. It was found that over a mean follow-up duration of 27 years, the mean individual cumulative external dose was 25 mSv between 1945 and 2005. The proportion of deaths attributable to external radiation exposure for this cohort was estimated to be approximately 1% of all deaths from solid cancer [21].

Although there is some evidence of increased cancer risk for low doses of radiation (< 100 mGy), it should be noted that at such low dose levels most studies do not have adequate power to enable precise risk estimation, especially when confounding factors such as cancer site and age at exposure are considered [22, 23]. Additionally, it can take many years for radiation-induced malignancies to appear; thus, patient age and life expectancy are important factors to include when analysing risk [24].

Even though there are no data relating directly to low-dose radiation risks in those with LS, there is evidence of CT associated malignancy in the general population. In the US, 0.9% of cancer cases could be attributed to diagnostic X-rays based on data from 1991 to 1996 [25], and it rises to as high as 2% by 2013 with increasing use of CT [15]. Overall, these data indicate a potentially small risk of malignancy induced by CT that could become significant with wider application of CT-based screening.

### Potential radiation risk of low dose CT based techniques

CT is routinely used for diagnosis, staging and surveillance of colorectal cancer patients. CTC has been developed over the last three decades as a safe and accurate CT based technique for CRC diagnosis with similar performance to colonoscopy [26]. While colonoscopy offers lesion biopsy and polypectomy, potential advantages of CTC include less invasiveness, less strong laxative bowel preparation, decreased procedural risks of perforation, rapid image acquisition and processing and potentially greater compliance [15, 27]. Nevertheless, CTC is not recommended for routine colonic surveillance in patients with LS [28], and colonoscopy is still preferred because molecular pathology in LS is different. The biological significance of smaller and flatter (or minimally protruding) polyps is considerably higher in LS patients [29], and for these small polyps the detection rate of CTC is not comparable to that in colorectal endoscopy [30]. Notwithstanding, colonoscopy is an imperfect surveillance test with annual CRC incident rates of between 1–3% according to Prospective Lynch Syndrome Database [31]. Therefore, CTC has a possible role as an adjunct to colonoscopy, as it permits complementary investigation of the colonic wall in sites that are more difficult to visualize at endoscopy, such as flexures. CTC also permits evaluation of the outer colonic wall and colonic mesentery; thus, theoretically increases the detection of early cancer. However, such a role would only be justified in LS patients if the risks of radiation associated harm were outweighed by the added benefits.

There are no data addressing the risk of CTC in LS patients. However, one study conducted in the general population showed the best estimated absolute lifetime cancer risk related to radiation exposure from CTC using 2005 scan parameters is about 0.14% for a 50-year-old, and approximately 0.07% for a 70-year-old. Using newer optimized CTC scanners and protocols, these values can be reduced further by a factor of 5 or 10 [32]. A risk/benefit analysis published in 2011 estimated a 0.15% risk of radiation-related cancer for an individual (from 50 to 80 years) undergoing CTC every 5 years [33]. These authors demonstrated that the benefits of CTC greatly outweigh any potential radiation-related risk and promulgated optimization of scan parameters to further reduce radiation exposure [15].

Standard CT scan protocols are used routinely in LS patients for surveillance and early detection of recurrent or metastatic tumor after successful treatment of colorectal cancer, with interval scans performed every 6 or 12 months and for a period of at least 3 years (www.nice.org.uk/guidance/ng151). Again, the risk/benefit of such a surveillance strategy in LS patients is not routinely considered in relation to the potential of excessive harm from radiation. This is particularly relevant given the large numbers of such scans performed each year and the possibility of using magnetic resonance imaging (MRI) as a non-IR alternative for surveillance in the future. Compared to CTC, the accuracy of MRI colonography is relatively low limiting its utility for adenoma screening [34].

### Potential risk of radiotherapy in LS patients

#### MMR gene mutation/down-regulation may be associated with previous radiotherapy and subsequent tumorigenesis

Radiation initiates a complex molecular network of DNA damage response (DDR) pathways and MMR proteins play important roles in DDR. Despite a paucity of evidence, radiotherapy may cause mutation or down-regulation of MMR genes and lead to further tumorigenesis. For example, increased risk of colorectal cancer has been reported in survivors of many types of cancer, such as Hodgkin's lymphoma, Wilms tumor, testicular and prostate cancer, bone cancer and central nervous system malignancies [35]. Survivors from Hodgkin's lymphoma treated with infradiaphragmatic radiotherapy had an increased risk (fivefold) of developing CRC. Compared with CRC in the general population, therapy related CRC showed more frequent loss of MSH2/MSH6 staining (13% vs 1%, *P* < 0.001) and a higher MSI frequency (24% vs 11%, *P* = 0.003) [35]. In a case report, a 74-year-old man with Muir-Torre syndrome (a variant/subtype of LS) and *MSH2* germline mutation was diagnosed with pleomorphic liposarcoma in a previous radiation field. IHC staining of this patient showed loss of MSH2 and MSH6 expression in the tumor [36]. Moreover, the clinical relevance and overall frequency of *MSH6* inactivating alterations in the development of glioblastomas have been investigated [37]. In this study, MSH6 protein expression was detected in all pre-treatment cases but was lost in 7 out of 17 recurrences from matched post-radiotherapy and chemotherapeutic agent treatment (41%, *P* = 0.016). Similarly, extensive loss of MSH6 expression (18%) was found common among colorectal carcinomas treated with neoadjuvant radiotherapy despite preserved pre-treatment staining and stable microsatellite [38]. These findings may suggest a novel association of somatic MMR gene alterations, such as mutation or epigenetic modification, with previous anticancer treatment.

#### Neoadjuvant radiotherapy

Neoadjuvant radiotherapy has been shown effective in reducing tumor burden and the use of neoadjuvant chemoradiotherapy (NCRT) is recommended for all newly diagnosed rectal adenocarcinoma with a clinical stage T3 or T4 [10]. However, a wide variation in the extent of radiation induced tumor regression was observed between individuals [39]. Complications, side effects, and toxicity from radiotherapy treatment of rectal cancer have been reported and the potential benefit must therefore be balanced against the risks [10].

It has been suggested that DNA MMR deficiency may indicate sensitivity to radiotherapy. For example, de Rosa et al. [40] reported that patients with d-MMR rectal cancer (n = 29) underwent Fluoropyrimidine-based neoadjuvant chemoradiation followed by surgery were associated with a pathologic complete response (pCR) rate of 27.6% compared to 18% among patients without LS. Similarly, Meillan et al. [41] reported that patients with d-MMR (n = 23) had higher pathologic downstaging rate, higher tumor regression grade, and a longer recurrence-free survival. More recently, d-MMR has been related to radio-resistance. In 2020, Ye et al. [42] reported that patients with d-MMR tumors (n = 66) who received NCRT achieved significantly worse disease-free survival (DFS) (*P* = 0.026) compared to those treated with neoadjuvant chemotherapy (NCT) alone, even though d-MMR was associated with improved DFS in patients receiving NCT (*P* = 0.034). On the contrary, NCRT improved DFS (*P* = 0.043) in patients with MMR proficient (p-MMR) tumors, especially for stage III cancer (*P* = 0.02). Another study published in 2020 evaluating pre-operative chemoradiotherapy in patients with locally advanced rectal cancer also indicated the potential chemo/radio-resistant role of d-MMR in rectal cancer [43]. In this study, 4450 MSI-negative and 636 MSI positive patients were treated with definitive chemoradiation followed by resection. The pCR rate was 8.9% for MSI negative and 5.9% for MSI positive patients. It should be noted that MSI positive status does not equal to MMR deficiency and therefore the lower pCR rate for MSI patients cannot be interpreted as resistance of d-MMR to radiotherapy. All these studies mentioned above used neoadjuvant chemoradiotherapy, no data were available to directly address the relationship between radio-sensitivity of d-MMR tumor and radiotherapy. These contradictory results may suggest the need for further research using radiotherapy alone, if possible, to clarify the radiation associated risks for MSI positive or d-MMR rectal cancer patients. The overall harm from neoadjuvant chemoradiotherapy may warrant the potential of using immunotherapy for locally advanced rectal cancers.

## Indirect studies for the association of LS with radio-sensitivity

With very little direct evidence of radiation associated risks in LS patients, assessments of potential harm from studies using cells, animal models and limited human sources are reviewed in this section.

### MMR in DNA damage response (DDR)

The DNA mismatch repair pathway is a highly conserved part of the DDR process active in the response to radiation-induced DNA damage as well as endogenous damage [44, 45]. It is involved in the removal of not only mismatched DNA pairs, small insertions and deletions arising during replication and recombination but also those caused by oxidative stress and some mutagens. Mutations inactivating this pathway are often associated with genomic instability and cancer predisposition [46].

Double-strand DNA breaks (DSBs) are the principle cytotoxic lesion for IR and is a well characterized key mediator of DNA damage. Defective DNA MMR may contribute to the instability of the genome by allowing the accumulation of genetic alterations that involve the pivotal components of the DDR pathways. For example, increased rates of mutations in MSI tumor cells have been reported to involve proteins essential for the recognition of DSBs and downstream signalling, such as ATM, MRE11 [47, 48], and DNA PKcs [49] required for non-homologous end joining. Interactions between the MMR proteins and some of the key DNA damage signalling molecules (e.g. ATR, ATM, Chk1 and Chk2) suggest that MMR proteins may play more direct roles in triggering a damage response. MMR proteins may also recruit damage signalling kinases to damaged DNA following lesion recognition [9]. Martin et al. [44] suggested that MMR proteins may recognize and bind to IR-induced DNA damage, promote a G_2_/M cell cycle arrest, interact with RAD51 recombination pathway, and ultimately lead to apoptosis. It was also suggested that MMR related radio-sensitivity may be dependent on the dose rate of the radiation used. Loss of MMR appears to be associated with radio-resistance following low dose-rate IR and radio-sensitivity following acute high dose-rate IR. Moreover, it is possible that depending on the extent of radiation-induced damage, DNA repair pathways contribute differently.

In relevance to this review, MSH2 forms heterodimers with MSH6/MSH3 and is involved in mismatch-pair recognition and initiation of repair; whereas MLH1 forms a heterodimer with PMS2 and has the function of an endonuclease [50]. Additionally, it has been revealed that MSH2 protein plays a role in the suppression of recombination by aborting strand exchange between divergent DNA sequences [51] as well as an early role in the cell-cycle arrest in response to various DNA damaging agents including IR [52]. MSH2 may also contribute to the processing of clustered DNA damage and the execution of IR induced apoptosis [53]. Furthermore, MSH2 may suppress homologous recombination (HR) via regulation of RAD51; therefore, for LS patients increased HR activity may result in increased resistance to radiotherapy and these resistant tumors may have increased rates of IR-induced genetic instability, elevated tumor heterogeneity and subsequently more malignant and invasive tumors [52]. In addition, *MLH1*-deficiency in human colon carcinoma (HCT-116) cells has been linked to ineffective G_2_/M checkpoint arrest following IR [54]. MLH1 protein may also have a role in suppressing IR-induced mitotic recombination stimulated by DSBs [55].

### Studies using primary cells or cell lines

#### G_2_ chromosomal radio-sensitivity

Chromosomal radio-sensitivity, manifested as an increased yield of chromatid aberrations when cells are exposed to IR during G_2_ phase of the cell cycle, is a well-known phenomenon in peripheral blood lymphocytes or fibroblasts from skin biopsies of patients with certain genetic disorders as well as cell lines derived from individuals with familial cancers of various types. It was hypothesized that persons at risk of developing a familial cancer might have inherited deficiency in one of their DNA repair systems and this might be reflected in G_2_ chromosomal radio-sensitivity [56]. However, an investigation using LS derived cell lines did not provide conclusive evidence. In this study, Franchitto et al. [57] used lymphoblastoid cell lines obtained from 3 controls and 7 LS patients carrying mutations either in *MLH1* (6 patients) or *MSH2* (1 patient) at the heterozygous state. Chromosome damage was induced by 0.5 Gy of X-ray [0.7 Gy/minute (min)] in synchronized G_2_ cells. It was found that G_2_ sensitivity in LS cells was not higher than that observed in control cells even though lymphoblasts from patients heterozygous for *MLH1* showed a higher yield of chromatid-type exchanges. The lack of G_2_ chromosomal sensitivity to IR was also observed in the lymphocytes of LS patients shown in a human study below. It is possible that cells with MMR genes in heterozygous state can still perform sufficient repair function.

#### Association of MMR proteins with radio-sensitivity

The roles that MMR proteins play in DDR in response to IR remain controversial and some of the conflicting results on the association of MMR proficiency with the radio-sensitivity of cells have been demonstrated and discussed by Martin et al. [44]. Briefly, radio-resistance of d-MMR cells was enhanced with low dose rate and was attributed to inefficient apoptotic signalling or loss of suppression of RAD51, an essential component in HR. Loss of MLH1 and MSH2 were also reported to be associated with reduced G_2_ /M arrest after IR with no effect on cell survival. Increased sensitivity of d-MMR cells to a number of DNA damaging agents, including high dose-rate IR, was related to inefficient early G_2_/M checkpoint and decreased DSB repair. In addition to the far different experimental settings between research laboratories, most of these studies used inaccurate outdated methods, such as the cell survival assay; and therefore, more accurate, state-of-the-art cellular and cytogenetic approaches are strongly proposed for updated knowledge in this area.

### Animal studies

It was found from the animal studies that at radiotherapy relevant high doses, IR can induce gastrointestinal or colorectal tumors in d-MMR mice as well as high levels of various types of mutation.

#### Radiation exposure accelerated intestinal tumor growth in *Mlh1*-knockout mice

Tokairin et al. [58] reported in 2006 that *Mlh1*-knockout mice spontaneously developed gastrointestinal tumors (GIT) and thymic lymphomas by 48 weeks of age. In their study, 2-week or 10-week old *Mlh1*^+/+^, *Mlh1*^+/−^ and *Mlh1*^−/−^ mice on a C57BL/6 background were exposed to whole-body X-irradiation at 2 Gy (0.7 Gy/min). It was found that irradiation accelerated GIT development in 10-week old *Mlh1*^−/−^ mice but had little effect at 2 weeks. In contrast, the vast majority of *Mlh1*^+/-^ and *Mlh1*^+/+^ mice were not susceptible to spontaneous or radiation-induced tumorigenesis until 72 weeks after birth. Thus, a potential elevated risk of secondary cancers should be considered for LS patients after radiotherapy.

#### The interplay of IR and inflammation in CRC pathogenesis in *Mlh1*-deficient mice

Inflammatory bowel disease frequently accompanied by silenced *Mlh1* gene plays a key role in the development of CRC [59]. In the study published by Morioka et al. [60] in 2015, *Mlh1*^−/−^ and *Mlh1*^+*/*+^ mice aged 2 weeks or 7 weeks were given a single whole-body X-irradiation of 2 Gy. At 10 weeks, some were treated with 1% dextran sodium sulphate (DSS) in drinking water for 7 days to induce mild inflammatory colitis. In *Mlh1*^+*/*+^ mice, no colon tumors were observed after radiation exposure with or without DSS treatment. DSS treatment alone triggered colon tumor development in *Mlh1*^*−/−*^ mice, and exposure to radiation prior to DSS treatment increased the number of tumors in these mice.

#### *Mlh1* deficiency and the risk of space radiation exposure

In 2019, Patel et al. [61] reported that age-related MMR deficiencies with accumulated MSI could lead to hematopoietic stem cell malignancy following radiation exposure. In this study, *Mlh1*^+*/+*^ and *Mlh1*^+*/−*^ mice harbouring MSI were exposed to 1 or 2.5 Gy of γ-rays and 0.1 or 1 Gy of ^56^Fe ion particles. It was found that allelic deficiency in *Mlh1* significantly increased the risk of hematopoietic malignancy, and the loss of *Mlh1* function was associated with high levels of single nucleotide mutations, insertions and deletions in resulting tumors. In contrast, tumorigenesis in *Mlh1*^+*/*+^ mice was not significantly increased in both types of radiation used. In addition, a significantly higher mean insertion and deletion size (≥ 5 and ≥ 10 base pairs) in all *Mlh1*^+*/−*^ cohorts compared to the *Mlh1*^+*/*+^ cohorts may indicate that *Mlh1* not only plays a role in mismatch repair but also in DSB repair.

### Human studies

#### The expression of MMR genes in high background radiation area

The city of Ramsar, in northern Iran, has the highest level of natural background radiation in the world (up to 260 mGy annually from radon exposure); however, research on the inhabitants of this area discovered no significant prevalence of radiation-related diseases or cancer compared to those in normal background areas [62]. One study published in 2019 [63] evaluated the expression of *MLH1* and *MSH2* genes among the inhabitants and the results showed a significant upregulation of *MLH1* in the residents compared to the control group; whilst *MSH2* expression showed no significant difference between these two groups. Additionally, the expression of both *MLH1* and *MSH2* was associated with age and gender as well as the length of residency in the area. The authors suggested the triggering of mismatch repair system by natural radiation which may be associated with hormesis effect and adaptive response.

#### Normal G_2_ chromosomal radio-sensitivity and cell survival in a LS family

In 1988, Bender et al. [56] analysed chromosome aberration yields induced by X-rays (0–8 Gy; 1 Gy/min) administered in G_2_ phase in skin fibroblasts and lymphocytes obtained from both affected and unaffected members of a LS family and found that these cells exhibited indistinguishable responses from normal controls. Again, the skin fibroblasts and lymphocytes are more likely to be heterozygous for the MMR genes, and the expected chromosomal aberrations probably can only be detected in homozygous cells and tumour cells.

## Other factors

As with many diseases, factors other than genetic predisposition can also complicate the IR associated radio-sensitivity in LS patients. For example, the CRC risk is reported in one study to be 96% in males and 39% in females with *MSH2* mutation. Extra-colonic cancer risk in *MSH2* deficient females and males was 69% and 34%, respectively. No difference in colorectal and extra-colonic cancer risks between *MLH1* deficient females and males was identified [64]. Age may also be an important factor as age-dependent increase in radio-sensitivity has been observed in *Mlh1*^−/−^ mice [58]. However, children under the age of 10 may be more radio-sensitive than older children based on the results of a cytogenetic analysis which demonstrated that after CT examination the frequencies of dicentrics and excess acentric fragments in blood lymphocytes were significantly increased for this age group [65].

## Discussion and conclusions

Low-dose IR is currently used routinely across the world for CT staging and surveillance of Lynch patients with CRC and yet there is no published information, guidance or recommendations available to inform clinicians, radiologists or patients about the relative risks in LS patients compared to sporadic CRC patients. In part this may be due to the contradictory evidence presented in this review which leads to no firm conclusions about the risks of low-dose IR. If IR can be confirmed relatively harmless, then the medical teams and patients can feel reassured to continue utilizing staging/surveillance CT; and the extending of CTC to the role as an adjunct to screening colonoscopy can also be investigated for future practice. However, there is no current data to support an increasing role for CTC in Lynch patients and more compelling evidence of safety would be required before this could be considered. Should low-dose IR be considered harmful then alternative methods for staging and surveillance of Lynch patients with CRC could be recommended, for example whole body MRI has been shown to be as accurate as CT in a large multicentre trial of colorectal cancer staging [66].

Similarly, oncologists routinely offer neoadjuvant radiotherapy preoperatively for Lynch patients with advanced rectal cancer frequently without knowing the MMR status of these patients in advance. Moreover, most oncologists will be unfamiliar with the evidence of risk/benefit when using high radiation dose treatment to the tumor and its surrounding pelvic tissues. Therefore, whether therapeutic doses of radiation can cause MMR mutation and lead to secondary tumorigenesis also require urgent investigation.

This review considers the literature associated with the potential radiation risks for LS patients. No direct evidence has been found for low-dose radiation risk of CT scans to high-risk patients with hereditary germline mutations, such as LS patients. Studies using LS associated primary cells or tumor cell lines with defective MMR genes at both low and high doses showed contradictory results in terms of cell radio-sensitivity after radiation exposure. However, there seems to be more evidence supporting relative radio-resistance and higher mutation rate for these cells. Results from animal studies showed elevated radiation risk for d-MMR mice potentially reducing the effectiveness of radiotherapy, worsening tumor prognosis and increasing the risk of new cancers in the surrounding tissues.

To date, the radio-sensitivity of primary cells or cell lines is mainly determined using the clonogenic survival assay or the expression of apoptotic markers. However, with ethical approval, it may be feasible and beneficial to study radio-sensitivity by directly analysing the peripheral blood lymphocytes and primary tumor cells from clinically and genetically confirmed LS patients using cytogenetic techniques to detect d-MMR associated chromosome and DNA damage induced by IR. For example, premature chromosome condensation coupled with fluorescence in situ hybridization [67] and γ-H2AX analysis [68, 69] have been developed more recently than those used in the earlier cytogenetic study on primary LS cells by Bender et al. in 1988 as discussed above. A strong case can be made for revisiting this topic using the newer assays that are highly sensitive and have been proven in the detection of chromosome damage after CT scans [70]. They may therefore be able to detect the chromosome aberrations that are unable to be detected using conventional cell survival assays or G_2_ assay at low doses.

It may also be beneficial to use genetically modified animal models or human cell lines with d-MMR and expose them to X-rays mimicking the doses of CT scans and radiotherapy. However, further considerations including the group sizes are needed to identify statistically significant effects following such low dose exposures. Furthermore, whether the presence of germline mutations may increase the risks of radiation toxicity or secondary malignancies is a major concern for clinicians. It would be beneficial to carry out retrospective studies to investigate the risk of somatic and germline MMR mutations and subsequent cancer rates and relate these results to previous CT surveillance and neoadjuvant radiotherapy in a large cohort of CRC patients, taking into consideration the MMR and MSI status, prognosis, gender, and age, etc.

Furthermore, quantification for mitochondrial DNA (mtDNA) mutations and deletions could represent a potential biomarker for radiation risks in LS patients. In 2020, Borghini et al. [71] reported that IR may lead to mtDNA mutations and content changes in cells, which are major driving mechanisms for vascular aging, neurodegeneration and carcinogenesis.

In conclusion, there is no current data addressing the risks of using IR for the diagnosis, staging, surveillance and treatment of LS patients and the existing knowledge in this area is outdated. Therefore, further research using the cutting-edge technologies is urgently required to provide the essential information for clinical guidance.

## Data Availability

Data sharing not applicable to this article as no datasets were generated or analysed during the current study.
